# Relationship between Thinking Dispositions, Working Memory, and Critical Thinking Ability in Adolescents: A Longitudinal Cross-Lagged Analysis

**DOI:** 10.3390/jintelligence12060052

**Published:** 2024-05-21

**Authors:** Shuangshuang Li, Ziyue Wang, Yijia Sun

**Affiliations:** Faculty of Education, Henan Normal University, Jianshe Road, Muye District, Xinxiang 453000, China; wangziyue20237@stu.htu.edu.cn (Z.W.); sunyijia20228@stu.htu.edu.cn (Y.S.)

**Keywords:** thinking dispositions, working memory, critical thinking ability, adolescents

## Abstract

Previous studies have demonstrated that thinking dispositions and working memory are closely related to students’ critical thinking ability. However, little is known about whether bidirectionality between thinking dispositions, working memory, and critical thinking ability exists in adolescence. This study, therefore, explored this aspect across two time points. Participants were 509 Chinese adolescents (mean age at Time 1 = 14.09 years; 59.7% girls). At Time 1, adolescents were administered the measures of thinking dispositions, working memory, and critical thinking ability. They were reassessed using these measures at Time 2 one year later. The results revealed a bidirectional longitudinal relationship between adolescents’ thinking dispositions and critical thinking ability, suggesting that thinking dispositions at Time 1 predicted critical thinking ability at Time 2; critical thinking ability at Time 1 also predicted subsequent thinking dispositions in adolescents. Furthermore, working memory at Time 1 showed a larger predictive effect on critical thinking ability at Time 2 compared with thinking dispositions at Time 1. These findings underscore the role of early thinking dispositions and working memory in promoting adolescents’ critical thinking ability.

## 1. Introduction

Critical thinking is widely regarded as a vital skill in the 21st century and has long been of interest in educational and psychological research (Santos-Meneses et al. 2023). Critical thinking ability enables students to achieve academic success, solve real-life problems, and function effectively in the modern world (Akpur 2020; Hwang et al. 2023; Ku et al. 2019; Vidal et al. 2023). Developing students’ critical thinking ability is not only essential for their social adjustment but also enhances the overall quality of their education. Therefore, an increasing number of countries worldwide have placed special importance on the cultivation of critical thinking abilities at all levels of education (Alpizar et al. 2022; Fan and See 2022; Hwang et al. 2023). Adolescence is a critical developmental stage of high-order thinking and a key period for cultivating critical thinking abilities (Alpizar et al. 2022; Lin and Shih 2022). A deeper understanding of the factors influencing adolescents’ critical thinking ability is undoubtedly important and can help in designing interventions to increase their capabilities. Previous studies have demonstrated that thinking dispositions and working memory are closely related to students’ critical thinking ability (e.g., Dwyer et al. 2014; Li et al. 2021). However, little is known about whether bidirectionality between thinking dispositions, working memory, and critical thinking ability is found in adolescence and how thinking dispositions and working memory might be associated with critical thinking ability across time. The current research aimed to examine the bidirectional associations among adolescents’ thinking dispositions, working memory, and critical thinking ability across two time points one year apart.

### 1.1. Thinking Dispositions and Critical Thinking Ability

Critical thinking is reasonable, reflective thinking focused on deciding what to believe or do (Ennis et al. 2005). This definition implies that critical thinking helps people engage less in cognitive bias and make better decisions in complex situations (Dwyer et al. 2014; Ennis 2018). In the context of theories of intelligence, critical thinking might be a lower-order factor below fluid intelligence as it strongly emphasizes evaluating information, reasoning logically, and forming judgments based on sound evidence (Carroll 1993; Dwyer et al. 2014; Ennis et al. 2005). Several researchers have proposed that personality factors, i.e., thinking dispositions, such as openness to ideas, typical intellectual engagement, and need for cognition, cause people to invest more effort in intellectual pursuits (Anglim et al. 2022; Cacioppo and Petty 1982; Fleischhauer et al. 2010; Goff and Ackerman 1992). Building on Cattell’s (1963) Investment Theory, intellectual effort is theorized to direct the application of one’s fluid intelligence and lead to the acquisition of knowledge. Thinking dispositions have emerged as important influences on intellectual activities alongside cognitive abilities because these dispositions concern not only what people are intellectually able to accomplish but also how they typically tend to invest their cognitive capacities (Fleischhauer et al. 2010; Perkins et al. 2000). These theoretical accounts of fluid intelligence suggest that thinking disposition might be an important predictor of individual differences in critical thinking.

Theoretical models related to critical thinking also suggest a substantial relationship between thinking dispositions and critical thinking ability (Dwyer et al. 2014; Facione 2015). For instance, the integrated critical thinking framework developed by Dwyer et al. (2014) suggests that thinking dispositions are crucial for critical thinking ability. Thinking disposition is often described as a consistent willingness, motivation, inclination, and intention to be engaged in thinking processes while reflecting on significant issues, making decisions, and solving problems (Ku and Ho 2010; Sosu 2013; Stanovich and Stanovich 2010). However, differences in theoretical conceptualizations and measurement have resulted in multiple definitions of thinking dispositions’ construct. For example, Facione and Facione (1992), drawing on the Delphi report, proposed seven dimensions of thinking dispositions such as open-mindedness and truth-seeking. In Perkins et al.’s (1993) conceptualization, thinking dispositions entail the tendency to be broad and adventurous, planful and strategic, intellectually careful, and metacognitive. Halpern (1998) identified that thinking dispositions encompass five dimensions: willingness to engage in and persist at a complex task, habitual use of plans and the suppression of impulsive activity, flexibility or open-mindedness, willingness to abandon nonproductive strategies in an attempt to self-correct, and awareness of social realities so that thoughts can become actions. Despite different models and theoretical structures of the thinking disposition, notions of openness and reflective skepticism pervade the different taxonomies (Sosu 2013). Therefore, the present study measured these two dimensions of thinking dispositions.

Dispositional influences on individual critical thinking ability are multifaceted. First, thinking dispositions affect the extent to which people are inclined to perform critical thinking skills such as analysis and evaluation (Stanovich and Stanovich 2010). Second, possessing a higher level of thinking dispositions might facilitate students to arrive at reasonable conclusions by considering different viewpoints and options (Ku and Ho 2010). Third, thinking dispositions encourage individuals to approach new information or viewpoints with a healthy dose of doubt and inquiry (Sosu 2013). A high level of thinking dispositions helps individuals become more aware of their own cognitive biases and less likely to make hasty judgments or fall victim to misleading or false information (Li et al. 2023). Facione (2015) also highlighted that thinking dispositions and critical thinking abilities are mutually reinforcing. That is, students with a higher level of thinking dispositions might have better critical thinking abilities, which may, in turn, improve their thinking dispositions and vice versa (Dwyer et al. 2017; van Rensburg and Rauscher 2022).

Previous empirical studies have shown evidence favoring that thinking disposition is closely related to critical thinking ability. Ku and Ho (2010) found that a higher level of thinking disposition significantly predicted university students’ higher critical thinking ability. Li et al. (2021) also revealed that thinking dispositions, including analyticity, self-confidence, and inquisitiveness, were positively related to critical thinking ability. Additionally, recent studies based on adult samples have investigated the relations of thinking dispositions with the ability to reason without being biased by prior beliefs, a crucial aspect of critical thinking (e.g., Ding et al. 2020; Li et al. 2023; Šrol and De Neys 2021; Schubert et al. 2021). The results from these studies offer sufficient evidence that there is a close link between thinking dispositions and critical thinking ability.

### 1.2. Working Memory and Critical Thinking Ability

Working memory refers to the cognitive system responsible for temporarily holding and manipulating information required for complex cognitive tasks (Baddeley 2021). The integrated critical thinking framework also emphasizes that working memory serves as a cognitive foundation for critical thinking ability (Dwyer et al. 2014). Both the storage and processing components of working memory are essential for the development of critical thinking abilities. First, critically thinking about information relies on processing that simultaneously actively keeps goal- or task-related representations in mind (Dwyer et al. 2014). Second, applying critical thinking to problem solving is also directly affected by a person’s ability to engage in a controlled, planful search of memory and effortful retrieval of additional goal- or task-related information as needed (Dwyer et al. 2014). Finally, working memory may play a pivotal role in inhibiting thinking bias and resolving the cognitive conflict involved in critical thinking ability (Bonnefon 2018; West et al. 2008). Thinking critically highlights adopting appropriate goals, resolving cognitive conflicts, and taking appropriate actions (Dwyer et al. 2014; West et al. 2008). Working memory as an ensemble of cognitive mechanisms enables individuals to monitor potential thinking bias and resolve cognitive conflict during the thinking process (Baddeley 2021; Cowan 2022). Accordingly, errors or mistakes in critical thinking might arise from lower-level working memory capacity or a failure of working memory.

Working memory has been shown to predict performance for fluid intelligence (Kyllonen and Christal 1990; Schneider and Niklas 2017; Wang et al. 2021). Meta-analytic studies have demonstrated that working memory and fluid intelligence share around 50% to 85% of their latent variance (Kane et al. 2005; Oberauer et al. 2005). These results imply that working memory is associated with critical thinking, which highlights the ability to solve complex problems by means of mental operations such as identifying relationships and drawing inferences (Dwyer et al. 2014; Ennis 2018). Empirical research has also indicated that working memory plays an important role in various aspects of critical thinking ability, such as deductive reasoning and the inhibition of thinking biases. For instance, Noone et al. (2016) found that working memory measured by tone monitoring and letter-memory tasks predicted critical thinking ability, including argument analysis, verbal reasoning, and hypothesis testing skills. Evidence from cognitive training has shown that adolescents’ deductive reasoning improved significantly after four weeks of working memory training (Ariës et al. 2016). A recent study by Li et al. (2021) showed that individual differences in working memory capacity significantly predicted performance in critical thinking tasks. Additionally, research has shown that working memory significantly contributes to adults’ ability to override the belief bias when completing syllogistic reasoning tasks (e.g., Ding et al. 2020; Schubert et al. 2021; Toplak et al. 2014).

### 1.3. The Present Study

The integrated critical thinking framework suggests that individual differences in critical thinking ability might arise because of individual differences in working memory (cognitive ability) or in thinking dispositions (habitual thinking tendency or style). However, the theory fails to clarify whether thinking dispositions and working memory play equally important roles in critical thinking ability. More empirical research is required to reveal how thinking dispositions and working memory jointly influence critical thinking. Therefore, the present study focused on how working memory and thinking dispositions are associated with critical thinking ability to further clarify the relative effects of cognitive and non-cognitive factors on critical thinking.

The definition of critical thinking emphasizes its cognitive attributes and highlights the importance of general abilities in this process, implying that the effect of working memory may be stronger than that of thinking dispositions. Indeed, previous research indicated working memory predicted critical thinking ability above and beyond thinking dispositions (Li et al. 2021). However, these studies, mostly using cross-sectional designs, were unable to clarify the direction and reciprocal relationship between thinking dispositions, working memory, and critical thinking ability. Therefore, longitudinal research is required to further check the stability and generalizability of the results regarding these relationships. In addition, little is known about how thinking dispositions and working memory contribute to the development of adolescents’ critical thinking abilities. Previous research has mostly focused on adult samples, and evidence from adolescents is relatively scarce. Considering that adolescence is a critical developmental stage during which critical thinking ability exhibits a prominent ascending trend (Lin and Shih 2022), students should be developing critical thinking ability in secondary school (Alpizar et al. 2022). Thus, understanding the role of thinking dispositions and working memory in adolescents’ critical thinking ability can aid in designing instructions for cultivating critical thinking. Such an investigation was expected to advance our knowledge regarding the role of thinking dispositions and working memory in critical thinking ability.

In summary, the purpose of the current study was to expand on the existing literature by examining the longitudinal and bidirectional relationships between thinking dispositions, working memory, and critical thinking ability among Chinese adolescents. To achieve this, the study employed a cross-lagged design and collected data on adolescents’ thinking dispositions, working memory, and critical thinking ability across two time points one year apart. Cross-lagged panel analysis is particularly relevant as it allows us to test the direction and strength of the link between thinking dispositions, working memory, and critical thinking ability while controlling for the prior levels of these three variables. Based on a synthesis of relevant theoretical perspectives and empirical research, we developed the following research hypotheses to guide our data analysis and interpretation of results: (a) thinking dispositions have a significant cross-lagged effect on critical thinking ability and (b) working memory has a larger cross-lagged effect on critical thinking ability compared with thinking dispositions.

## 2. Methods

### 2.1. Participants

Participants were recruited from two schools in Henan Province, a central province in China. Students participated in the initial data collection at Time 1 (T1) and in the second data collection at Time 2 (T2), 12 months later. A total of 601 adolescents participated in the study at T1, with 92 adolescents lost at T2 (15.3% attrition), mainly because of missing the assessment or being transferred to other schools. The final sample size comprised 509 participants who completed the measurements in the study (59.7% girls; age: *M* = 14.09 years, *SD* = 1.61 at T1). There were no significant differences between the included and excluded adolescents in any of the variables. At T1, the distribution of subjectively reported household income (1 = “very poor” to 5 = “very rich”) was as follows: 1.6% very rich, 2.6% rich, 70.7% middle income, 16.5% poorer, and 8.6% very poor. This study was approved by the Institutional Review Board of the corresponding authors’ university. Informed consent was obtained from all participants and their parents and teachers.

### 2.2. Measures

#### 2.2.1. Thinking Dispositions

The Chinese version of the Critical Thinking Disposition Scale (CTDS; Sosu 2013) was employed to assess thinking dispositions. The 11-item CTDS was presented on a five-point, Likert-type scale ranging from 1 (strongly disagree) to 5 (strongly agree). Seven items assessed openness, which reflected the tendency to be actively open to new ideas (e.g., “I am often on the lookout for new ideas”), and four items measured reflective skepticism, conveying the tendency to learn from one’s past experiences and be questioning of evidence (e.g., “I often re-evaluate my experiences so that I can learn from them”). The Chinese version of the CTDS has good reliability and construct validity (Yang 2016). The total score for the CTDS was obtained by summing all item responses. Higher scores indicate a higher level of thinking disposition. The Cronbach’s alphas were 0.88 and 0.85 at T1 and T2, respectively. We also constructed the measurement model of thinking dispositions; the fit statistics of this model were acceptable: *χ*^2^/*df* = 2.24, CFI = 0.95, TLI = 0.92, RMSEA = 0.049, and SRMR = 0.041.

#### 2.2.2. Working Memory

The modified running memory task based on Akira Miyake et al. (2000) was employed to assess working memory. Participants were presented with a series of digits, with list lengths varying between 5, 7, 9, and 11. The task required the participants to recall the last four digits presented in the list. Each digit was presented for 1000 ms. The interval between any two digits was 100 ms. The four list lengths were varied randomly across trials to ensure that participants continuously updated their working memory representations until the end of each trial. The task comprised five practice trials and 24 test trials (six trials within each list length). The number of correctly recalled trials was recorded. The Cronbach’s alphas were 0.69 and 0.73 at T1 and T2, respectively.

#### 2.2.3. Critical Thinking Ability

The Chinese version of the Cornell Critical Thinking Test-Level X (CCTT-X, Ennis et al. 2005) was used to measure adolescents’ critical thinking ability. The CCTT-X is suitable for students in grades 4–14 and the Chinese version of the CCTT-X has good reliability and construct validity (Bi et al. 2019; Kwan and Wong 2014). Therefore, we employed this test to tap into Chinese adolescents’ critical thinking ability. The CCTT-X describes a fictitious situation followed by a series of alternative inferences and conclusions from which participants must choose. It comprises 71 multiple-choice items and measures different aspects of critical thinking ability: inductive reasoning, deductive reasoning, judging observations and credibility, and identifying assumptions. Each question has three response options, and only one is correct. The total CCTT-X score was computed by summing the scores on each item. Higher scores indicate a higher level of critical thinking ability. The Cronbach’s alphas were 0.71 and 0.68 at T1 and T2, respectively.

### 2.3. Procedure

At both T1 and T2, the assessment measures of critical thinking ability, thinking dispositions, and working memory were administered by professionally trained graduate students in a quiet room. Before the critical thinking ability test began, the researchers provided a detailed explanation of the task to ensure that the adolescents understood the task process. One assessment session lasted approximately 60 min, and adolescents were given a break between tasks. Participants reported their age, gender, and household income via questionnaires at T1. All tests were completed during regular school hours.

### 2.4. Statistical Analysis

The preliminary descriptive analyses used SPSS 25.0 to calculate the correlation between the main study variables. A cross-lagged panel model examining the bidirectionality between adolescents’ working memory, thinking dispositions, and critical thinking ability across two time points was estimated using Mplus 7.4, statistically controlling for adolescents’ age, gender, and household income. Model fit was evaluated by the ratio of chi-square to degrees of freedom (*χ*^2^/*df*), comparative fit index (CFI), Tucker–Lewis index (TLI), root mean square error of approximation (RMSEA), and standardized root mean square residual (SRMR). The acceptable fit indices were χ^2^/*df* ≤ 5, CFI ≥ 0.90, TLI ≥ 0.90, RMSEA ≤ 0.08, and SRMR ≤ 0.08.

## 3. Results

### 3.1. Preliminary Analyses

We conducted paired samples t-tests to compare the difference between measures at T1 and T2. The results indicated that critical thinking ability (*t* = 10.79, *p* < 0.001), thinking dispositions (*t* = 8.02, *p* < 0.001), and working memory (*t* = 10.53, *p* < 0.001) at T2 were significantly higher than at T1. Table 1 displays the means, standard deviations, and correlation coefficients of the major variables at T1 and T2. Critical thinking ability was significantly positively correlated with working memory and thinking dispositions at concurrent time points. Working memory was significantly positively correlated with thinking dispositions at concurrent time points. All cross-lagged correlations between the three variables were significant and positive, suggesting that there was indeed a positive relationship between working memory, thinking dispositions, and critical thinking ability. We conducted partial correlation analyses between three measures at T2 while controlling for these measures at T1. The results indicated that critical thinking ability at T2 was still significantly correlated with working memory (*r* = 0.12, *p* < 0.01) and thinking dispositions (*r* = 0.10, *p* < 0.05). In contrast, there was no significant correlation between working memory and thinking dispositions after controlling for these measures at T1.

### 3.2. Cross-Lagged Path Analysis

**Figure 1:**
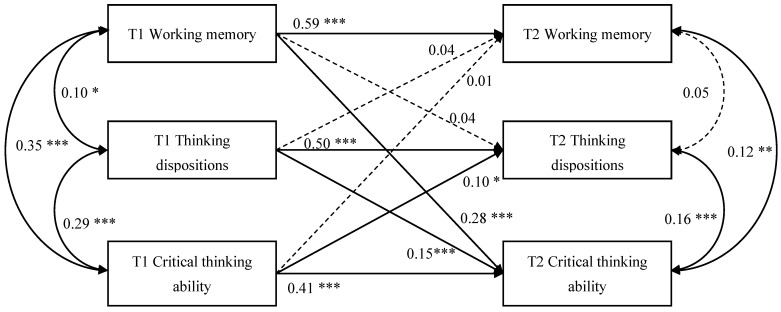
Cross-lagged panel model of adolescents’ working memory, thinking dispositions, and critical thinking ability across Time 1 and Time 2. Note: Although covariates (gender, age, and household income) were controlled for every study variable, the paths are not shown for conciseness. * *p* < 0.05, ** *p* < 0.01, *** *p* < 0.001.

A cross-lagged model was established to examine the predictive effect of variables at T1 on those at T2 after controlling for age, gender, and household income (Figure 1). The fit indices indicated that the model performed excellently: *χ*^2^/*df* = 1.07, CFI = 0.99, TLI = 0.99, RMSEA = 0.012, and SRMR = 0.018. In combination, predictors explained the following percentage of variance in the measures at T2: 34.7% for working memory, 37.7% for thinking dispositions, and 39.7% for critical thinking ability. All three measures were significantly (*p* ≤ 0.05) correlated with each other at T1 (Figure 1). At T2, when previous associations were controlled for, only two associations remained significant (working memory–critical thinking ability and thinking dispositions–critical thinking ability; Figure 1).

As shown in Figure 1, the autoregressive paths of working memory (*β* = 0.59, *p* < 0.001), thinking dispositions (*β* = 0.50, *p* < 0.001), and critical thinking ability (*β* = 0.41, *p* < 0.001) were all significant. Three of the cross-lagged paths were significant (see Figure 1). Specifically, the cross-lagged path from T1 working memory to T2 critical thinking ability was significant (*β* = 0.28, *p* < 0.001). However, we did not find a significant association between T1 critical thinking ability and T2 working memory. This means that working memory can predict critical thinking ability but not vice versa. The cross-lagged paths from T1 thinking dispositions to T2 critical thinking ability (*β* = 0.15, *p* < 0.001) and from T1 critical thinking ability to T2 thinking dispositions (*β* = 0.10, *p* = 0.016) were significant. This indicates a bidirectional link between critical thinking ability and thinking dispositions. Furthermore, we conducted the Wald test (Muthén and Muthén 2017) to examine whether the cross-lagged effects of working memory and thinking dispositions at T1 on critical thinking ability at T2 differed significantly. The results indicated that T1 working memory had a larger effect on T2 critical thinking ability than T1 thinking dispositions, *χ*^2^(1) = 7.27, *p* = 0.007.

## 4. Discussion

The present study aimed to provide additional evidence for the role of working memory and thinking disposition in critical thinking ability. Longitudinal empirical evidence was required, as most studies have relied on cross-sectional designs (e.g., Ding et al. 2020; Li et al. 2023; Šrol and De Neys 2021; West et al. 2008). Additionally, as most previous studies were conducted on adults, we provided additional evidence with adolescents. Focusing on this sample was important given that adolescence is a critical developmental stage for high-order thinking and a pivotal period in the cultivation of critical thinking ability (Alpizar et al. 2022; Lin and Shih 2022). To this end, we examined the relationship between working memory, thinking dispositions, and critical thinking ability using a two-wave cross-lagged design among a sample of adolescents to provide an in-depth understanding of how working memory and thinking dispositions are associated with critical thinking ability across time. We found that after controlling for other demographic variables, thinking dispositions and critical thinking ability had a bidirectional predictive relationship. The cross-lagged model also demonstrated that adolescents’ early working memory exerted a larger effect on later critical thinking ability than did early thinking disposition. The present findings expand existing studies by demonstrating the facilitative role of working memory and thinking dispositions in adolescents’ development of critical thinking ability.

Importantly, our findings first employed a cross-lagged design to demonstrate the bidirectional relationships between thinking dispositions and adolescents’ critical thinking ability. Specifically, early thinking disposition is a predictor of adolescents’ later critical thinking, and early critical thinking ability predicts their subsequent thinking disposition. The results provide direct evidence supporting the theoretical view that thinking dispositions and critical thinking ability may contribute to each other over time (Facione 2015; van Rensburg and Rauscher 2022). Thinking critically about specific information requires a good attitude toward different and opposing views, willingness to use critical thinking skills, and habitual engagement in critical thinking; thus, strong thinking dispositions can benefit critical thinking ability (Dwyer et al. 2014; Ku and Ho 2010). Moreover, children with good critical thinking ability are more likely to have advanced thinking skills, such as recognizing assumptions, deductions, interpretations, and analysis, helping them participate in more complex cognitive processing activities and gradually improving their thinking dispositions (Facione 2015). In this vein, adolescents with higher levels of thinking dispositions can develop more critical thinking skills and have higher critical thinking ability, which may further foster the development of their thinking dispositions. In recent years, researchers have designed intervention programs for cultivating good thinkers, suggesting that critical thinking ability and thinking dispositions mutually enhance each other (Dwyer et al. 2017). The research results indicate that thinking dispositions and critical thinking abilities may be mutually influential. While training thinking abilities in educational practice, it is important to emphasize the cultivation of middle school students’ thinking dispositions to promote their simultaneous development.

The present results suggest that adolescents’ early working memory is predictive of their critical thinking ability one year later, with corresponding autoregressive effects and demographic variables controlled. This finding is consistent with the integrated critical thinking framework, suggesting that working memory may serve as a cognitive foundation to foster high-order cognitive processes, that is, critical thinking (Dwyer et al. 2014). Our results are also consistent with those of previous cross-sectional studies (Ding et al. 2020; Li et al. 2023), demonstrating that working memory is associated with the sub-skills of critical thinking ability such as deductive reasoning and inhibition of thinking bias. Applying critical thinking to solve problems is directly affected by a person’s ability to actively maintain goal- and task-related information in mind (Dwyer et al. 2014; Noone et al. 2016). Working memory processing also contributes to critical thinking ability by monitoring and suppressing the activation of unwanted information or biased thinking (West et al. 2008). Working memory directly facilitates the processing and manipulation of information essential for critical thinking tasks such as evaluating and synthesizing complex ideas (Baddeley 2021; Dwyer et al. 2014). It allows individuals to hold multiple pieces of information simultaneously, enabling them to make connections and draw conclusions effectively. Working memory enables individuals to consider multiple perspectives and hold various ideas in mind, which is crucial for critical thinking (Cowan 2022; Dwyer et al. 2014). This ability to maintain and manipulate diverse information enhances the depth and accuracy of critical thinking. Furthermore, the current study indicates that, compared with thinking dispositions, early working memory shows a larger predictive effect on later critical thinking ability. This echoes theoretical perspectives that highlight the underlying cognitive characteristics of critical thinking (e.g., Dwyer et al. 2014; Facione 2015) and is also in line with existing experimental studies suggesting that working memory measures predicted critical thinking ability over and above thinking dispositions (Li et al. 2021).

The results also depict that working memory may be mainly determined by the prior level of working memory (as reflected by the strong autoregressive path) but not previous critical thinking ability. Perhaps the stability of working memory in the present study is so strong that there may not be enough variance left over for critical thinking to predict, which leads to the non-significance of the cross-lagged paths from critical thinking to working memory. Nevertheless, future studies with three or more time points are necessary to assess the directionality of these relationships to further investigate the directions of effect and the associations between working memory and critical thinking ability. Future research may also consider including adolescents in a wider age range to examine whether age or class levels may moderate the relationships.

Some limitations of the present study should be considered. The first set of limitations relates to the measures. We employed only one task or scale to measure thinking dispositions, working memory, and critical thinking ability owing to time restrictions during data collection. This prevented us from using confirmatory factor analysis to alleviate the task-impurity problem embedded in a single measure. Therefore, the cross-lagged model might have limited stability. A wide range of measures for thinking dispositions, working memory, and critical thinking ability are recommended to further improve the generalization of the findings. Although these tests employed in the present study have good psychometric characteristics and are appropriate for Chinese participants (Bi et al. 2019; Yang 2016; Zhao et al. 2022), there is also the possibility of cultural differences in measures of working memory, thinking dispositions, and critical thinking ability. That is, cultural factors should be considered in future studies. For example, future research could carry out a worldwide survey and consider using more inclusive assessments to tap into cognitive ability (Gutchess and Rajaram 2023; Holden and Tanenbaum 2023; Wang 2021). Another limitation relates to the measure of the control variable. The robustness of the study results can be increased by controlling for meaningful covariates such as metacognition or logical knowledge structures in future research (Akcaoğlu et al. 2023; Raoelison et al. 2021). Finally, cross-lagged longitudinal analysis can provide some support for causal inferences but does not allow us to definitively infer causality. Future studies using quasi-experimental intervention design are required to validate the causal relationships between thinking dispositions, working memory, and critical thinking ability.

Despite these limitations, the findings of the current study have relevant implications for theoretical advancement. Although theories such as the integrated critical thinking framework suggest that thinking dispositions and working memory may be recruited in applying critical thinking skills, no theory has explicitly specified how thinking dispositions and working memory relate to critical thinking ability and the relative contributions of thinking dispositions and working memory to the development of adolescents’ critical thinking ability. Our results suggest that working memory is more strongly associated with adolescents’ later critical thinking abilities compared with thinking dispositions. This result highlights that, compared to non-cognitive factors, cognitive factors might play a more significant role in critical thinking ability. Future theoretical models of critical thinking could consider more clearly stating the importance of cognitive abilities, such as working memory. The results practically highlight the importance of early thinking dispositions and working memory in the development of critical thinking ability and suggest the utility of improving adolescents’ thinking dispositions to foster their critical thinking ability.

## Figures and Tables

**Table 1:** Mean (M), standard deviation (SD), and bivariate correlations of study variables.

	*M*	*SD*	1	2	3	4	5	6	7	8
1. Gender	-	-	-							
2. Age	14.09	1.61	−0.25 **	-						
3. Household income	2.71	0.72	0.03	−0.05	-					
4. T1 Working memory	17.28	5.23	0.05	0.12 **	0.11 **	-				
5. T1 Thinking dispositions	40.99	5.58	0.02	0.04	0.05	0.10 *	-			
6. T1 Critical thinking ability	40.41	6.35	0.08	−0.03	0.06	0.35 ***	0.29 ***	-		
7. T2 Working memory	19.67	5.97	0.06	0.03	0.08	0.59 ***	0.10 *	0.21 ***	-	
8. T2 Thinking dispositions	43.06	6.37	0.02	0.11 **	0.14 *	0.22 ***	0.53 ***	0.24 ***	0.18 **	-
9. T2 Critical thinking ability	43.31	6.56	0.12 **	−0.05	0.04	0.39 ***	0.30 ***	0.56 ***	0.31 ***	0.30 ***

Note. T1, Time 1; T2, Time 2. * *p* < 0.05, ** *p* < 0.01, *** *p* < 0.001.

## Data Availability

The data that support the findings of this study are available from the corresponding author upon reasonable request.

## References

[B1-jintelligence-12-00052] Akcaoğlu Mustafa Öztürk, Mor Ezgi, Külekçi Erkan (2023). The mediating role of metacognitive awareness in the relationship between critical thinking and self-regulation. Thinking Skills and Creativity.

[B2-jintelligence-12-00052] Akpur Uğur (2020). Critical, reflective, creative thinking and their reflections on academic achievement. Thinking Skills and Creativity.

[B3-jintelligence-12-00052] Alpizar David, Vo Thao, French Brian F., Hand Brian (2022). Growth of critical thinking skills in middle school immersive science learning environments. Thinking Skills and Creativity.

[B4-jintelligence-12-00052] Anglim Jeromy, Dunlop Patrick D., Wee Serena, Horwood Sharon, Wood Joshua K., Marty Andrew (2022). Personality and intelligence: A meta-analysis. Psychological Bulletin.

[B5-jintelligence-12-00052] Ariës Roel J., Ghysels Joris, Groot Wim, van den Brink Henriette Maassen (2016). Combined working memory capacity and reasoning strategy training improves reasoning skills in secondary social studies education: Evidence from an experimental study. Thinking Skills and Creativity.

[B6-jintelligence-12-00052] Baddeley Alan D. (2021). Developing the concept of working memory: The role of neuropsychology. Archives of Clinical Neuropsychology.

[B7-jintelligence-12-00052] Bi Jinggang, Dong Yuqi, Han Ying (2019). Cujin pipanxingsiwei fazhan de zaixianxuexi huodongmoxingsheji yanjiu [Design of online learning activities model promoting critical thinking]. Chinese Journal of Distance Education.

[B8-jintelligence-12-00052] Bonnefon Jean-François (2018). The pros and cons of identifying critical thinking with system 2 processing. Topoi.

[B9-jintelligence-12-00052] Cacioppo John T., Petty Richard E. (1982). The need for cognition. Journal of Personality and Social Psychology.

[B10-jintelligence-12-00052] Carroll John B. (1993). Human Cognitive Abilities: A Survey of Factor-Analytic Studies.

[B11-jintelligence-12-00052] Cattell Raymond B. (1963). Theory of fluid and crystallized intelligence: A critical experiment. Journal of Educational Psychology.

[B12-jintelligence-12-00052] Cowan Nelson (2022). Working memory development: A 50-year assessment of research and underlying theories. Cognition.

[B13-jintelligence-12-00052] Ding Daoqun, Chen Yang, Lai Ji, Chen Xiyou, Han Meng, Zhang Xianyi (2020). Belief bias effect in older adults: Roles of working memory and need for cognition. Frontiers in Psychology.

[B14-jintelligence-12-00052] Dwyer Christopher P., Hogan Michael J., Stewart Ian (2014). An integrated critical thinking framework for the 21st century. Thinking Skills and Creativity.

[B15-jintelligence-12-00052] Dwyer Christopher P., Hogan Michael J., Harney Owen M., Kavanagh Caroline (2017). Facilitating a student-educator conceptual model of dispositions towards critical thinking through interactive management. Educational Technology Research and Development.

[B16-jintelligence-12-00052] Ennis Robert H. (2018). Critical thinking across the curriculum: A vision. Topoi.

[B17-jintelligence-12-00052] Ennis Robert H., Millman Jason, Tomko Tomas N. (2005). Cornell Critical Thinking Tests, Level X and Level Z Manual.

[B18-jintelligence-12-00052] Facione Peter A., Facione Noreen C. (1992). California Critical Thinking Disposition Inventory.

[B19-jintelligence-12-00052] Facione Peter A. (2015). Critical Thinking: What It Is and Why It Counts.

[B20-jintelligence-12-00052] Fan Keji, See Beng H. (2022). How do Chinese students’ critical thinking compare with other students? A structured review of the existing evidence. Thinking Skills and Creativity.

[B21-jintelligence-12-00052] Fleischhauer Monika, Enge Sören, Brocke Burkhard, Ullrich Johannes, Strobel Alexander, Strobel Anja (2010). Same or different? Clarifying the relationship of need for cognition to personality and intelligence. Personality and Social Psychology Bulletin.

[B22-jintelligence-12-00052] Goff Maynard, Ackerman Phillip L. (1992). Personality-intelligence relations: Assessment of typical intellectual engagement. Journal of Educational Psychology.

[B23-jintelligence-12-00052] Gutchess Angela, Rajaram Suparna (2023). Consideration of culture in cognition: How we can enrich methodology and theory. Psychonomic Bulletin & Review.

[B24-jintelligence-12-00052] Halpern Diane F. (1998). Teaching critical thinking for transfer across domains: Dispositions, skills, structure training, and metacognitive monitoring. American Psychologists.

[B25-jintelligence-12-00052] Holden LaTasha R., Tanenbaum Gabriel J. (2023). Modern assessments of intelligence must be fair and equitable. Journal of Intelligence.

[B26-jintelligence-12-00052] Hwang Jihyun, Hand Brian, French Brian F. (2023). Critical thinking skills and science achievement: A latent profile analysis. Thinking Skills and Creativity.

[B27-jintelligence-12-00052] Kane Michael J., Hambrick David Z., Conway Andrew R. A. (2005). Working memory capacity and fluid intelligence are strongly related constructs: Comment on Ackerman, Beier, and Boyle. Psychological Bulletin.

[B28-jintelligence-12-00052] Ku Kelly Y. L., Ho Irene T. (2010). Dispositional factors predicting Chinese students’ critical thinking performance. Personality and Individual Differences.

[B29-jintelligence-12-00052] Ku Kelly Y. L., Kong Qiuyi, Song Yunya, Deng Lipeng, Kang Yi, Hu Aihua (2019). What predicts adolescents’ critical thinking about real-life news? The roles of social media news consumption and news media literacy. Thinking Skills and Creativity.

[B30-jintelligence-12-00052] Kwan Yee Wan, Wong Angela F. L. (2014). The constructivist classroom learning environment and its associations with critical thinking ability of secondary school students in Liberal Studies. Learning Environments Research.

[B31-jintelligence-12-00052] Kyllonen Patrick C., Christal Raymond E. (1990). Reasoning ability is (little more than) working-memory capacity?!. Intelligence.

[B32-jintelligence-12-00052] Li Shuangshuang, Ren Xuezhu, Schweizer Karl, Brinthaupt Thomas M., Wang Tengfei (2021). Executive functions as predictors of critical thinking: Behavioral and neural evidence. Learning and Instruction.

[B33-jintelligence-12-00052] Li Shuangshuang, Sun Yijia, Yang Huimin (2023). The influence of thinking dispositions on belief-bias inhibition process: Evidence from ERPs and neural oscillations. Thinking Skills and Creativity.

[B34-jintelligence-12-00052] Lin Wei-Lun, Shih Yi-Ling (2022). Developmental trends of different creative potentials in relation to adolescents’ critical thinking abilities. Thinking Skills and Creativity.

[B35-jintelligence-12-00052] Miyake Akira, Friedman Naomi P., Emerson Michael J., Witzki Alexander H., Howerter Amy, Wager Tor D. (2000). The unity and diversity of executive functions and their contributions to complex “frontal lobe” tasks: A latent variable analysis. Cognitive Psychology.

[B36-jintelligence-12-00052] Muthén Linda K., Muthén Bengt O. (2017). Mplus User’s Guide.

[B37-jintelligence-12-00052] Noone Chris, Bunting Brendan, Hogan Michael J. (2016). Does mindfulness enhance critical thinking? Evidence for the mediating effects of executive functioning in the relationship between mindfulness and critical thinking. Frontiers in Psychology.

[B38-jintelligence-12-00052] Oberauer Klaus, Schulze Ralf, Wilhelm Oliver, Süß Heinz-Martin (2005). Working memory and intelligence–their correlation and their relation: Comment on Ackerman, Beier, and Boyle 2005. Psychological Bulletin.

[B39-jintelligence-12-00052] Perkins David N., Jay Eileen, Tishman Shari (1993). Beyond abilities: A dispositional theory of thinking. Merrill-Palmer Quarterly.

[B40-jintelligence-12-00052] Perkins David, Tishman Shari, Ritchhart Ron, Donis Kiki, Andrade Al (2000). Intelligence in the wild: A dispositional view of intellectual traits. Educational Psychology Review.

[B41-jintelligence-12-00052] Raoelison Matthieu, Boissin Esther, Borst Grégoire, Neys Wim De (2021). From slow to fast logic: The development of logical intuitions. Thinking & Reasoning.

[B42-jintelligence-12-00052] Santos-Meneses Luis F., Pashchenko Taras, Mikhailova Aleksandra (2023). Critical thinking in the context of adult learning through PBL and e-learning: A course framework. Thinking Skills and Creativity.

[B43-jintelligence-12-00052] Schneider Wolfgang, Niklas Frank (2017). Intelligence and verbal short-term memory/working memory: Their interrelationships from childhood to young adulthood and their impact on academic achievement. Journal of Intelligence.

[B44-jintelligence-12-00052] Schubert Anna-Lena, Ferreira Mário B., Mata André, Riemenschneider Ben (2021). A diffusion model analysis of belief bias: Different cognitive mechanisms explain how cognitive abilities and thinking styles contribute to conflict resolution in reasoning. Cognition.

[B45-jintelligence-12-00052] Sosu Edward M. (2013). The development and psychometric validation of a Critical Thinking Disposition Scale. Thinking Skills and Creativity.

[B46-jintelligence-12-00052] Stanovich Keith E., Stanovich Paula J., Preiss David D., Sternberg Robert J. (2010). A framework for critical thinking, rational thinking, and intelligence. Innovations in Educational Psychology: Perspectives on Learning, Teaching and Human Development.

[B47-jintelligence-12-00052] Šrol Jakub, De Neys Wim (2021). Predicting individual differences in conflict detection and bias susceptibility during reasoning. Thinking and Reasoning.

[B48-jintelligence-12-00052] Toplak Maggie E., West Richard F., Stanovich Keith E. (2014). Rational thinking and cognitive sophistication: Development, cognitive abilities, and thinking dispositions. Developmental Psychology.

[B49-jintelligence-12-00052] van Rensburg Joalise J., Rauscher Willem (2022). Strategies for fostering critical thinking dispositions in the technology classroom. International Journal of Technology and Design Education.

[B50-jintelligence-12-00052] Vidal Sofia, Pereira Armanda, Núñez José C., Vallejo Guillermo, Rosendo Daniela, Miranda Sara, Tortella Jussara, Rosário Pedro (2023). Critical thinking predictors: The role of family-related and motivational variables. Thinking Skills and Creativity.

[B51-jintelligence-12-00052] Wang Qi (2021). The cultural foundation of human memory. Annual Review of Psychology.

[B52-jintelligence-12-00052] Wang Tengfei, Li Chenyu, Ren Xuezhu, Schweizer Karl (2021). How executive processes explain the overlap between working memory capacity and fluid intelligence: A test of process overlap theory. Journal of Intelligence.

[B53-jintelligence-12-00052] West Richard F., Toplak Maggie E., Stanovich Keith E. (2008). Heuristics and biases as measures of critical thinking: Associations with cognitive ability and thinking dispositions. Journal of Educational Psychology.

[B54-jintelligence-12-00052] Yang Zhibing (2016). Characteristic Analysis of Misinformation and Its Influence Factors for Transmission Intention. Unpublished. Ph.D. dissertation.

[B55-jintelligence-12-00052] Zhao Xin, Wang Yixuan, Maes Joseph H.R. (2022). The effect of working memory capacity and training on intertemporal decision making in children from low-socioeconomic-status families. Journal of Experimental Child Psychology.

